# No Effect of Anodal Transcranial Direct Current Stimulation Over the Motor Cortex on Response-Related ERPs during a Conflict Task

**DOI:** 10.3389/fnhum.2016.00384

**Published:** 2016-08-05

**Authors:** Alexander C. Conley, W. R. Fulham, Jodie L. Marquez, Mark W. Parsons, Frini Karayanidis

**Affiliations:** ^1^Functional Neuroimaging Laboratory, School of Psychology, Faculty of Science and IT, University of NewcastleNewcastle, NSW, Australia; ^2^Priority Research Centre for Stroke and Brain Injury, University of NewcastleNewcastle, NSW, Australia; ^3^Hunter Medical Research InstituteNewcastle, NSW, Australia; ^4^School of Health Sciences, Faculty of Health, University of NewcastleNewcastle, NSW, Australia; ^5^School of Medicine and Public Health, Faculty of Health, University of NewcastleNewcastle, NSW, Australia

**Keywords:** transcranial direct current stimulation, event-related potential, contingent negative variation, lateralized readiness potential, P300, ageing

## Abstract

Anodal transcranial direct current stimulation (tDCS) over the motor cortex is considered a potential treatment for motor rehabilitation following stroke and other neurological pathologies. However, both the context under which this stimulation is effective and the underlying mechanisms remain to be determined. In this study, we examined the mechanisms by which anodal tDCS may affect motor performance by recording event-related potentials (ERPs) during a cued go/nogo task after anodal tDCS over dominant primary motor cortex (M1) in young adults (Experiment 1) and both dominant and non-dominant M1 in older adults (Experiment 2). In both experiments, anodal tDCS had no effect on either response time (RT) or response-related ERPs, including the cue-locked contingent negative variation (CNV) and both target-locked and response-locked lateralized readiness potentials (LRP). Bayesian model selection analyses showed that, for all measures, the null effects model was stronger than a model including anodal tDCS vs. sham. We conclude that anodal tDCS has no effect on RT or response-related ERPs during a cued go/nogo task in either young or older adults.

## Introduction

Research into the potential merits of anodal transcranial direct current stimulation (tDCS) for therapeutic interventions in both motor (e.g., stroke) and psychological (e.g., depression) conditions is increasing, reflecting a desire to gain a greater understanding of the method by which tDCS elicits change in the neocortex. In this article, we utilize the high temporal resolution of event-related brain potentials (ERPs) to identify the mechanisms by which anodal tDCS over the motor cortex may affect response processes in healthy young and older adults.

tDCS involves the application of a weak current across the surface of the cortex via scalp electrodes (Nitsche and Paulus, 2000; Utz et al., 2010). When applied over the motor cortex, this current generates changes to motor output (Nitsche and Paulus, 2000). The nature of these changes is dependent on the positioning of stimulation and reference electrodes. Positive or anodal tDCS over the primary motor cortex (M1) increases the amplitude of motor-evoked potentials (MEPs) elicited by transcranial magnetic stimulation (TMS) pulses, whereas negative or cathodal tDCS reduces MEP amplitude (Nitsche and Paulus, 2001; Nitsche et al., 2003a; Utz et al., 2010). Functionally, the application of anodal tDCS over M1, has been shown to improve performance on motor control tasks. For instance, after receiving anodal tDCS over the M1, both young and old adults exhibited faster completion of the Jebsen Taylor Hand Function Test (JTT, Jebsen et al., 1969) which assesses performance of a number of functional upper limb movements (Boggio et al., 2006; Hummel et al., 2010). Improvements have also been shown on a range of cued movement tasks. Anodal tDCS over the dominant M1 resulted in faster and more accurate responses on sequential tapping tasks (Nitsche et al., 2003b; Vines et al., 2006, 2008). Healthy young adults also showed improved skill acquisition on a visually-directed pinch task following consecutive daily sessions of anodal tDCS over the M1 (Reis et al., 2009; Schambra et al., 2011). This improved speed and/or accuracy of motor performance following the application of anodal tDCS over the M1 in healthy adults is thought to be consistent with improved efficiency of motor pathways (Jacobson et al., 2012). Such findings have motivated the use of anodal tDCS over M1 as a rehabilitation tool in pathologies characterized by motor dysfunction (for a review see Flöel, 2014). Encouraging findings show that the application of anodal tDCS over M1 may restore some motor functioning in patients suffering from Parkinson’s disease (Fregni et al., 2006), dystonia (Benninger et al., 2011) and following a severe neurological trauma such as a stroke (O’Shea et al., 2014).

However, this rush to endorse anodal tDCS as a neurological intervention may be premature. A number of recent studies have failed to find a beneficial effect of anodal tDCS over M1 on performance in either young or old adults. On choice reaction time tasks, studies have shown no performance improvement following anodal tDCS compared to sham in either young adults (Pellicciari et al., 2013) or old adults (Lindenberg et al., 2013). Using a cued go/nogo task, Conley et al. (2015) found no impact of anodal tDCS over dominant M1 on response speed for either the dominant or the non-dominant hand in healthy young adults. That these null findings have all been evidenced using attention-driven response paradigms indicates that anodal tDCS may fail to enhance communication between the prefrontal cortex (PFC) and the primary and secondary motor areas. Investigation into the mechanisms by which anodal tDCS over M1 affects motor processes is therefore essential to establish the efficacy of anodal tDCS as a potential therapeutic intervention tool.

It is also important to examine the effectiveness of anodal tDCS on these response processes in healthy older adults. It is well known that healthy ageing is associated with gradual alterations to both cortical structure and functioning (Buckner, 2004; Raz et al., 2005; Seidler et al., 2010) as well as a reduction in processing speed (Salthouse, 2000). This decline in processing speed is associated with decreased behavioral performance and changes in ERP waveforms in older compared to young adults (Polich, 1997; Sterr and Dean, 2008; Ren et al., 2013). As most clinical neurological disorders that are likely to benefit from motor cortex tDCS interventions emerge in older adults, it is imperative to investigate the effects of stimulation in older adults, as they provide a much more appropriate baseline for clinical studies than do young adults.

As conventional behavioral measures, such as mean response times (RT) and error rates, represent the endpoint of decision making, they do not offer direct insight into the temporal evolution of attentional and motor processes that lead up to a response. The excellent temporal resolution of event-related potentials (ERPs) offers the capability to measure these processes and may therefore identify effects of anodal tDCS even in the absence of an overt behavioral effect. The few studies that have examined the effects of tDCS on ERPs have not investigated motor processes (Kongthong et al., 2013; Lafontaine et al., 2013; Lapenta et al., 2014). The only study to examine changes to ERP morphology following anodal tDCS over M1, measured TMS-elicited ERP rather than task-driven ERP components associated with stimulus and response processing (Pellicciari et al., 2013). Thus, it is still unclear whether tDCS over M1 affects the morphology or timing of ERP components associated with response processes.

A number of ERP components are associated with motor processes. The contingent negative variation (CNV) is a slow negative deflection that indexes processes associated with preparation of a motor response (Rockstroh et al., 1989). It typically emerges after a warning stimulus (cue) heralds the occurrence of an imperative stimulus (target) to which the participant must respond (Walter et al., 1964; Leuthold et al., 2004). Cues that provide valid information about the response required to the upcoming target generate a larger CNV compared to neutral cues (Leuthold and Schröter, 2011). CNV amplitude is indicative of level of motor preparation, with larger CNV being associated with faster responding. The CNV is associated with increased activation in both the M1 and the supplementary motor area (SMA, Gomez et al., 2003). Response selection and activation processes are indexed by the lateralized readiness potential (LRP). The LRP is a large negative deflection that indicates greater activation over the motor cortex of the hand associated with a correct response (Coles, 1989). When time-locked to the onset of the target (tLRP), it represents pre-motoric processes leading up to response selection. When time-locked to the onset of the response (rLRP), it represents motor processes leading from response selection to response execution (Masaki et al., 2004). The LRP is associated with activation at M1 and SMA, consistent with a role in motor planning and execution (Praamstra et al., 1996).

ERPs can be used to differentiate between motor and non-motor effects of anodal tDCS stimulation. This is particularly important because, although anodal tDCS over M1 is intended to stimulate the M1, stimulation may spread to other cortical areas depending on electrode size and location of the reference electrode (Miranda et al., 2013). In order to show specific effects of tDCS stimulation on motor processes, it is necessary to show that it specifically affects ERP components associated with response processes (e.g., CNV, LRP) and not ERP components associated with sensory and attention processes, such as the target-locked P300 (Picton, 1992; Linden, 2005; Polich, 2007). The P300 is a parietal positive peak that peaks at least 300 ms after the presentation of a task-relevant stimulus. P300 amplitude varies with task difficulty and target information, and its peak latency represents completion of stimulus evaluation. Functionally, the P300 is associated with activation in different cortical areas depending on the stimulus modality. Auditory stimuli elicit increased cortical activation in the inferior temporal cortex, whereas visual stimuli increase cortical activity at the posterior parietal cortex (Bledowski et al., 2004; Linden, 2005). These three ERP components can be thus used to measure attentional and motor processes that contribute to the timing and accuracy of a motor response.

**Figure 1:**
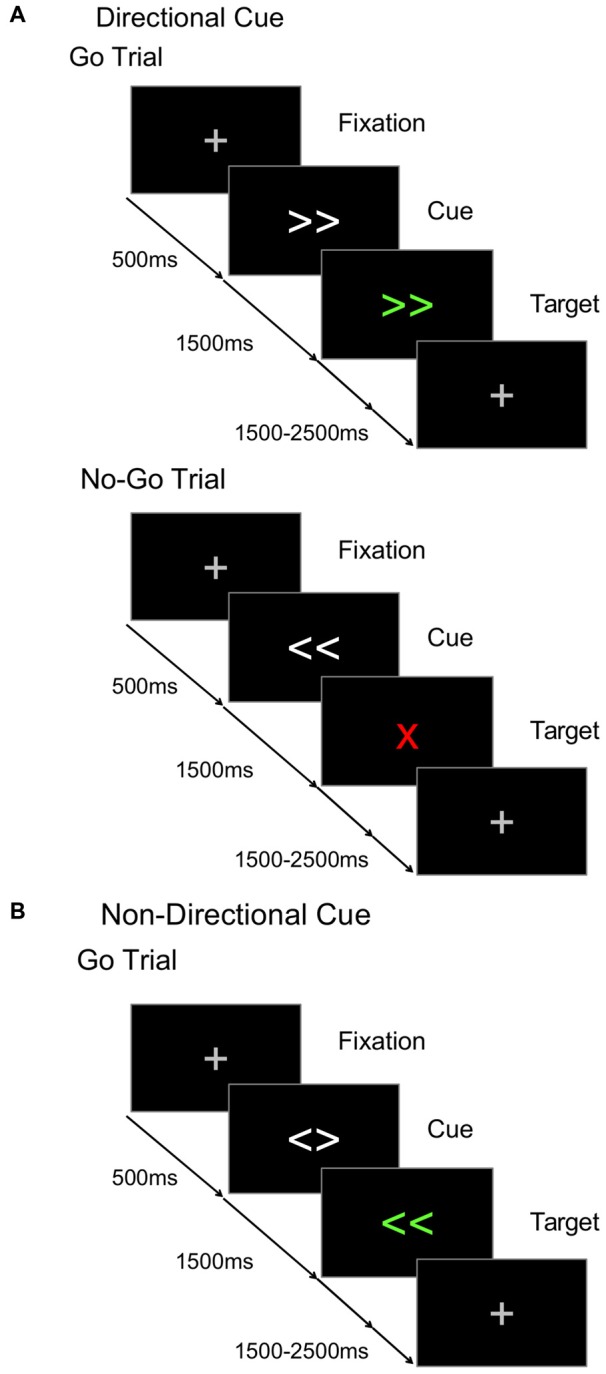
**Cued go/nogo task.** The time course for **(A)** go and nogo trials in directional cue condition, and **(B)** go trials in non-directional cue condition.

In this study, we examined whether anodal tDCS over the dominant or the non-dominant M1 produces selective changes to motor processes, as evidenced by response-related ERP components during a cued go/nogo task. The effects of anodal tDCS over M1 were examined in two experiments: one in healthy young and the other in healthy older adults. The cued go/nogo paradigm was used to elicit both motor and non-motor ERP components in order to test whether effects of anodal tDCS over M1 were specific to motor processes (Figure 1). This paradigm was selected because it manipulates the timing of response preparation processes by altering the contextual information given by the visual cue. Lapenta et al. (2014) examined the effects of anodal tDCS on ERPs elicited on a go/nogo task, but stimulation was applied over the dorsolateral PFC (DLPFC), rather than the motor cortex.

In the cued go/nogo task used here, some blocks used *directional cues* (Figure 1A) that provided valid information about whether the target would require a left or a right hand response, allowing preparation of the response required after target onset. Other blocks used *non-directional cues* (Figure 1B) that provided valid information about the timing of the upcoming target, but not its direction (Figure 1B). Thus participants could anticipate target onset but not prepare a left or right motor response. During the cue-target interval (CTI), directional cues were expected to elicit a larger CNV than non-directional cues, indicating the anticipatory preparation of the motor response. The efficiency of target processing can be assessed in the peak amplitude of the P300 (Kutas et al., 1977). Directional cues were expected to elicit a smaller P300 component compared to non-directional cues. As noted above, the target-locked LRP (tLRP) indexes response selection and the response-locked LRP (rLRP) is linked to response activation. Directional cues were expected to elicit an earlier tLRP and shorter duration of the rLRP compared to the non-directional cues, as greater preparation requires less effort to select and execute the appropriate response.

## Experiment 1

In Conley et al. (2015), we showed that anodal tDCS stimulation over the dominant M1 in young adults had no effect on behavioral response speed during a cued go/nogo task delivered during, immediately after or shortly after stimulation. Here, we examine whether this stimulation may have had an effect on ERP components representing response processes that led up to the motor response and that were not captured by RT.

Specifically, anodal tDCS over M1 could be expected to improve response preparation, resulting in larger CNV compared to the sham condition. It could facilitate response selection or response activation, reducing tLRP onset latency or rLRP duration, respectively. Finally, given evidence that anodal tDCS over M1 may have corollary effects on adjoining frontal and parietal areas (Miranda et al., 2013) involved in stimulus evaluation and context updating, it could impact amplitude and/or latency of the target-locked P300 component in either direction—either improving or reducing efficiency of attentional processes. As anodal tDCS was delivered over the dominant M1, any effects were expected to be greater over the dominant hemisphere.

## Method

### Participants

Twenty-four healthy young adults completed active and sham stimulation sessions^1^. One participant was removed from the analyses due to excessive artifact in their electrophysiological recording, so the remaining analysis was performed on 23 participants (9 males, mean age 21.2 ± 2.5 years). All participants were right handed as measured by the Edinburgh Handedness Inventory (Oldfield, 1971). All participants were screened for non-suitability for DCS, including epilepsy, major heart condition or any neurological implants.

The protocol was approved by the Hunter New England Human Research Ethics Committee (H-2013-0115), and was in accordance with the Declaration of Helsinki. All participants provided written informed consent prior to commencing the experiment.

### Transcranial Direct Current Stimulation Settings

Anodal tDCS stimulation was delivered by a battery-driven constant-current stimulator (neuroConn GmbH, Germany) and involved the application of a 1 mA current continuously for 20 min (with 10 s ramp-up/down at the beginning and end of the intervention) using two rubber electrodes (35 cm^2^) soaked in saline. The current density of the electrodes was 28.6 μA/cm^2^. The anode was placed over the left M1, while the cathode was placed over the supraorbital region of the contralateral hemisphere. Electrode placement on the scalp over the hand area of M1 was determined using the scheme for placement of the C3 EEG electrode according to the International 10/20 system, as used in Bachmann et al. (2010). This montage has previously been shown to be effective at increasing the excitability of the dominant M1 (Nitsche and Paulus, 2000, 2001). The sham stimulation condition involved the application of a 1 mA current for 50 s (10 s ramp up and 40 s application) followed by 20 min delay to match the duration of the active stimulation session. Stimulation conditions (active or sham) were assigned a code by one experimenter. Another experimenter who was blind to the correspondence between codes and stimulation condition entered the code during the experimental session. Thus neither participant nor the experimenter running the session was aware of the stimulation condition applied. Order of active and sham stimulation conditions was counterbalanced between subjects. Sessions were scheduled at least 3 weeks apart to avoid any carryover effects of stimulation.

### Cued Go/Nogo Paradigm

The task consisted of a S1-S2 trial sequence, where the cue (S1) validly predicted the onset of the target (S2) after a fixed CTI (1500 ms, Figure 1). Each trial began with a fixation cross (500 ms) that was replaced by the cue (1500 ms) which was, in turn, replaced by the target. Directional and non-directional cues were presented in separate randomized blocks. On non-directional cue blocks (Figure 1B), the cue consisted of two white arrows pointing in different directions (i.e., < >), and validly predicted the timing of target onset. The target was two green directional arrows (i.e., ≪, ≫) that specified a compatible left or right hand response. On directional cue blocks, the cue consisted of the same two white arrows, but now they pointed in the same direction (i.e., ≫ or ≪) validly predicting the green target. However, on 30% of trials, the target was a “nogo” stimulus (i.e., a red X) indicating that a response must be withheld. So, on informative cue blocks, participants could use the cues to prepare a left or right hand response, but had to await target onset to check whether the response must be withheld. On both directional and non-directional cue blocks, the target remained visible for 1000 ms and the subsequent target-cue interval was jittered (mean 2000 ms, random sequence, 1500–2500 ms). Participants completed five blocks of 80 trials (two blocks of non-directional and three blocks of directional cue conditions) and were instructed about the significance of non-informative and informative cues. Prior to testing on each session, participants completed two practice blocks (30 trials/block): one for each cue type.

### Procedure

In the first session, participants provided informed consent and completed a medical screening form and the Edinburgh Handedness Inventory. Prior to the administration of anodal tDCS or sham, participants completed the Grooved Pegboard Test (Schmidt et al., 2000) with left and right hands. The stimulation electrodes were then applied and participants received 20 min of either active or sham stimulation. Following stimulation, participants repeated the Grooved Pegboard Test and completed the Digit Span Test (forward, backward, ascending), the Trail Making Test (Tombaugh, 2004) and practice on the experimental task. The results of these tests are discussed in Conley et al. (2015). After EEG was set up, participants completed the experimental blocks of the cued go/nogo paradigm. EEG testing commenced approximately 40 min after termination of stimulation. At the completion of this session, participants were given a short questionnaire assessing their subjective comfort during the intervention, and were asked whether they thought they had received anodal tDCS or sham. Participants returned 3 weeks later to complete the second session, in which they received the other stimulation intervention.

### EEG Recording and Processing

Electrophysiological data was continuously sampled from 64 scalp electrodes at 2048 Hz/channel reference free using a Biosemi ActiView II system. Vertical and horizontal electro-oculogram (EOG) was recorded from the lateral, supra-orbital and infra-orbital electrodes of each eye. Continuous EEG files were referenced offline to average mastoids and filtered using a 0.02–30 Hz bandpass filter and a 50 Hz notch filter to remove line noise. EEG data were processed and analyzed using EEG Display 6.3.12 (Fulham, 2012).

Target-locked ERP waveforms were derived from 3000 ms epochs extracted from 300 ms prior to fixation onset to 800 ms after target onset. Separate waveforms were derived for go trials under each cue condition, for each hand and stimulation condition. CNV amplitude was measured at Cz as the mean amplitude over 1300–1500 ms post-cue (i.e., 200 ms prior to target onset) using a 200 ms baseline preceding the onset of the fixation point. Peak amplitude of the target-locked P300 was measured at Pz over 200–500 ms after target onset, relative to a 200 ms pretarget baseline in order to take variability in CNV into account.

tLRP and rLRPs were extracted from the C3/C4 electrode pair using the averaging method explained by Coles (1989):

(1) $$LRP\;=\;\left[Mean{\left(C4-C3\right)}_{LH\;responses}+Mean{\left(C3-C4\right)}_{RH\;responses}\right]/2$$

Both target- and response-locked waveforms were filtered using a 30 Hz zero-phase, low pass filter to reduce high frequency noise. tLRP waves were baselined across 200 ms prior to the onset of the target, whereas rLRP waves were baselined between 500 and 700 ms prior to the overt response. Onset latencies were extracted using 25% fractional area latency, which used the mean amplitude across the defined window as the threshold. The windows used were between 100–600 ms post-target onset for tLRPs and over 300–100 ms before the response for rLRPs.

### Data Analyses

Both the RT and the mean ERPs for go trials were analyzed using a repeated measures generalized linear model with three within-subjects factors: Stimulation (active, sham), Cue (directional, non-directional) and Response Hand (left, right). As accuracy was very high, these scores were not analyzed statistically. LRP analysis included only the Stimulation and Cue factors.

## Results

### Behavioral Results

**Figure 2:**
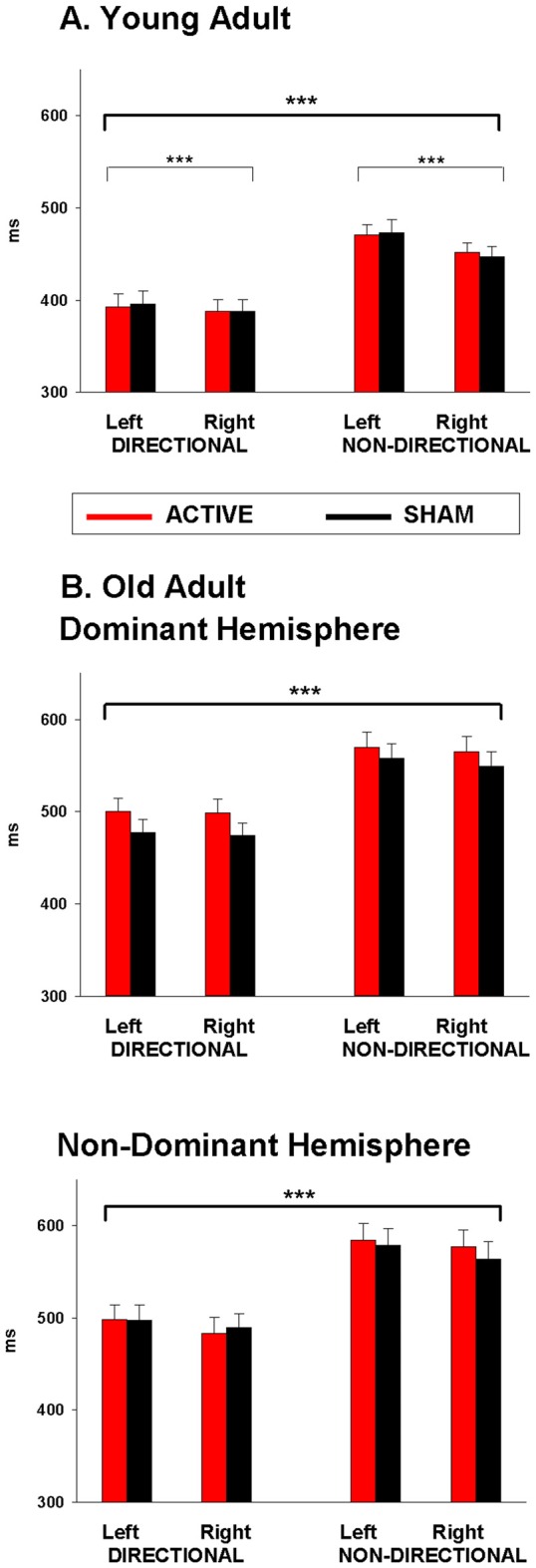
**Mean response time (RT) following anodal tDCS (red) and sham (black) for (A) Young adults over dominant hemisphere, and (B) Old adults over dominant hemisphere and non-dominant hemisphere.** Significant main effects are represented by asterisks (***p < 0.001).

Mean RTs for young adults are displayed in Table 1 (top). As shown in Figure 2A, young adults responded faster to directional than to non-directional cues (*F*_(1,22)_ = 149.86, *p* < 0.001) and with their right than their left hand (*F*_(1,22)_ = 20.63, *p* < 0.001). The right hand advantage was greater for non-directional cues (*F*_(1,22)_ = 10.17, *p* = 0.004). There was no effect of anodal tDCS on response speed (tDCS: 425.7 ± 11.7 ms, Sham: 426.2 ± 11.7 ms; *F*_(1,22)_ < 1).

**Table 1:** **Mean response time (RT, milliseconds) for young and old adults for each cue and hand following anodal tDCS and sham**.

Group/Stimulation	Directional left	Directional right	Non-directional left	Non-directional right
**Young adult**
Active	392.8 (14.0)	388.0 (12.7)	470.5 (11.1)	451.6 (10.5)
Sham	396.1 (13.8)	388.1 (12.0)	473.3 (13.8)	447.2 (11.0)
**Old adult**
*Dominant*
Active	500.5 (14.4)	499.0 (14.8)	570.2 (16.2)	565.1 (16.4)
Sham	477.3 (14.3)	474.6 (12.9)	558.1 (15.9)	549.1 (16.3)
*Non-Dominant*
Active	497.8 (16.5)	483.3 (16.9)	584.1 (18.5)	576.7 (18.8)
Sham	497.7 (16.3)	489.6 (14.7)	578.7 (18.2)	563.5 (18.7)

*Standard error of the mean is in parentheses*.

### Electrophysiological Results

**Figure 3:**
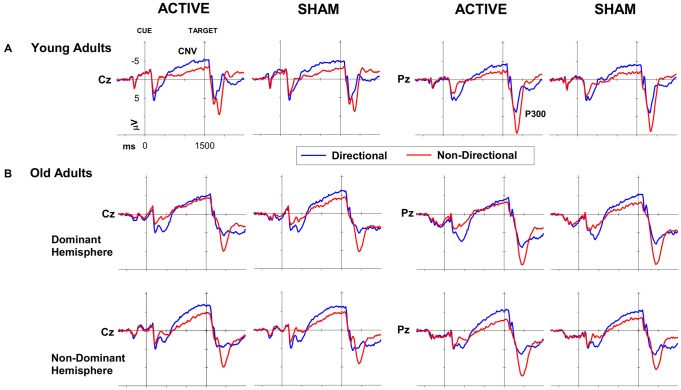
**ERP waveforms for directional (blue) and non-directional (red) conditions at Cz (left) and Pz (right) following active (i.e., anodal tDCS) and sham stimulation for (A) young and (B) old adults**.

Mean amplitudes for CNV and P300 ERP components for young adults are displayed in Tables 2, 3 (top), respectively. Peak P300 latencies are displayed for young adults in Table 4 (top). Figure 3A shows cue-locked ERP waveforms at Cz and Pz electrodes following anodal tDCS and sham stimulation. The CNV emerged around 500 ms post-cue onset, peaking just before target onset. CNV amplitude was larger for directional than non-directional cues (−4.9 vs. −2.8 μV; *F*_(1,22)_ = 18.9, *p* < 0.001) but did not vary with response hand (*F* < 1). As shown in Figure 3A, stimulation did not affect CNV amplitude or interact with cue or response hand (all *F* < 1).

**Table 2:** **Mean contingent negative variation (CNV) amplitude at Cz (microvolts) for young and old adults for each cue and hand following anodal tDCS and sham**.

Group/Stimulation	Directional left	Directional right	Non-directional left	Non-directional right
**Young adult**
Active	−5.0 (0.9)	−5.0 (0.9)	−3.1 (0.8)	−2.8 (0.7)
Sham	−5.3 (0.97)	−4.2 (1.0)	−2.9 (0.5)	−2.6 (1.0)
**Old adult**
*Dominant*
Active	−5.4 (1.1)	−4.0 (1.0)	−4.2 (0.9)	−3.9 (1.0)
Sham	−5.5 (0.97)	−6.1 (0.97)	−3.9 (1.3)	−4.1 (0.9)
*Non-Dominant*
Active	−7.4 (1.2)	−6.4 (1.2)	−4.5 (1.0)	−4.5 (1.2)
Sham	−5.8 (1.1)	−6.8 (1.1)	−4.9 (1.4)	−4.3 (1.1)

*Standard error of the mean is in parentheses*.

**Table 3:** **Peak P300 amplitude at Pz (microvolts) for young and old adults for each cue and hand following anodal tDCS and sham**.

Group/Stimulation	Directional left	Directional right	Non-directional left	Non-directional right
**Young adult**
Active	13.8 (1.0)	15.1 (1.1)	17.9 (1.1)	18.9 (0.9)
Sham	14.6 (1.1)	15.0 (1.1)	17.7 (1.0)	17.9 (1.1)
**Old adult**
*Dominant*
Active	15.0 (1.2)	15.0 (1.2)	19.0 (1.4)	17.6 (1.4)
Sham	15.9 (1.1)	15.1 (1.1)	19.3 (1.3)	18.3 (1.3)
*Non-Dominant*			
Active	13.9 (1.4)	14.5 (1.4)	17.2 (1.6)	15.9 (1.6)
Sham	13.9 (1.2)	13.8 (1.3)	15.5 (1.5)	14.9 (1.5)

*Standard error of the mean is in parentheses*.

**Table 4:** **P300 peak latencies (milliseconds) at Pz for young and old adults for each cue and hand following anodal tDCS and sham**.

Group/Stimulation	Directional left	Directional right	Non-directional left	Non-directional right
**Young adult**
Active	342.4 (16.9)	337.45 (18.5)	344.2 (10.6)	357.4 (6.9)
Sham	317.1 (14.3)	336.7 (15.9)	345.0 (14.3)	344.8 (10.4)
**Old adult**
*Dominant*
Active	465.6 (22.1)	470.0 (22.6)	442.4 (12.7)	448.2 (12.5)
Sham	469.5 (24.6)	432.7 (22.9)	452.9 (11.3)	470.0 (12.7)
*Non-Dominant*
Active	420.4 (25.3)	429.3 (25.9)	447.7 (14.5)	465.0 (14.3)
Sham	424.4 (28.2)	431.7 (26.3)	468.6 (13.0)	454.4 (14.6)

*Standard error of the mean is in parentheses*.

A large P300 emerged parietally following target onset. P300 amplitude was smaller for directional than non-directional cues (10.9 vs. 16.2 μV; *F*_(1,22)_ = 36.4, *p* < 0.001) and marginally for left than right hand responses (*F*_(1,22)_ = 5.2, *p* = 0.03). There was no effect of cue or response hand on P300 latency (both *p* > 0.3). There was no effect of stimulation on P300 amplitude or latency (all *p* > 0.05).

**Figure 4:**
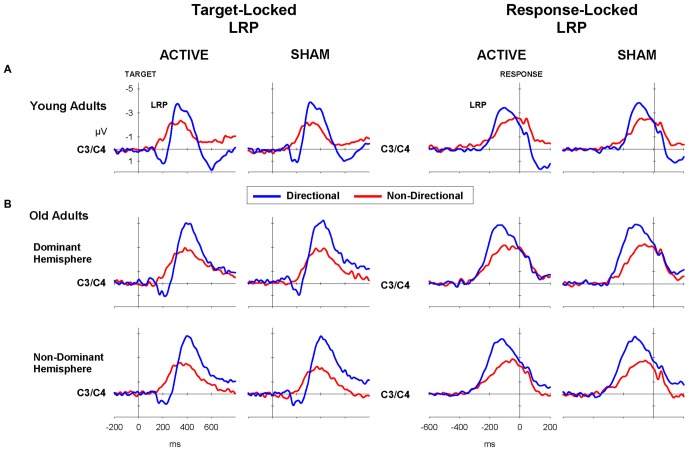
**LRP waveforms for directional (blue) and non-directional (red) conditions in target-locked (left) and response-locked (right) LRP waveforms following active (i.e., anodal tDCS) and sham for (A) young and (B) old adults**.

Mean tLRP and rLRP onset latencies for young adults are displayed in Table 5 (top). As shown in Figure 4A (left), tLRP emerged earlier for directional than for non-directional cues, with the latter showing an early positive dip (cue: 271 vs. 313 ms; *F*_(1,22)_ = 38.0, *p* < 0.001). rLRP had a later onset for directional than non-directional cues (Figure 4A, right; −109 ms vs. −131 ms; *F*_(1,22)_ = 25.4, *p* < 0.001). Stimulation did not significantly affect the onset latency of either tLRP or rLRP (all *F* < 1).

**Table 5:** **Mean onset latencies (milliseconds) for target-locked (tLRP) and response-locked (rLRP) for young and old for each cue and hand following anodal tDCS and sham**.

Group/Stimulation	Directional	tLRP Non-directional	Directional	rLRP Non-directional
**Young adult**
Active	273.8 (10.3)	312.9 (7.4)	−107.7 (8.5)	−134.0 (9.1)
Sham	268.1 (10.0)	313.3 (6.9)	−109.2 (9.1)	−128.6 (7.8)
**Old adult**
*Dominant*
Active	335.1 (13.9)	376.9 (6.8)	−130.6 (12.5)	−166.2 (10.8)
Sham	348.1 (11.1)	371.9 (7.7)	−106.0 (7.6)	−159.0 (9.9)
*Non-Dominant*
Active	335.4 (15.9)	387.4 (7.8)	−115.8 (14.3)	−165.1 (12.4)
Sham	341.7 (12.7)	389.1 (8.8)	−122.0 (8.8)	−163.6 (11.4)

*Standard error of the mean is in parentheses*.

## Discussion

Overall, behavioral and electrophysiological findings show that participants completed the task as expected, preparing their response to directional cues and waiting for target onset before responding for both cue types. The CNV was larger for directional cues that allowed response preparation, whereas the P300 was larger for non-directional cues that required greater post-target processing. LRPs also indicated that prepared responses showed earlier response selection (tLRP) and faster response activation (rLRP, Wild-Wall et al., 2003). Interestingly, despite the simple nature of the task, response selection for the non-directional cues showed a large “dip” in the tLRP, suggesting at least partial preparation of both responses in the CTI. This is likely to account at least partly for the RT delay for directional vs. non-directional cue blocks.

Despite the fact that the task showed strong behavioral and ERP effects in the expected direction, there was no evidence of any effect of anodal tDCS over M1 on any of the measures. As the sample consisted of healthy young adults, and stimulation was applied to M1 corresponding to their dominant hand, it is possible that the lack of any effect of anodal tDCS is due to a ceiling effect that precluded any further improvement (Wu and Hallet, 2005).

## Experiment 2

In Experiment 2, we examined whether there are beneficial effects of anodal tDCS to the dominant or non-dominant motor cortex on response processes in older healthy adults. Ageing is associated with reduced processing speed (Ren et al., 2013), as well as changes to the morphology of ERP waveforms associated with cognitive and response processes (Cespón et al., 2013). Compared to young adults, old adults tend to show slower stimulus evaluation, as evidenced by increased P300 latency across the lifespan (for a review see Polich, 1996). Old adults also show slower response selection (tLRP) and response activation (rLRP) processes compared to young adults (Yordanova et al., 2004; Kolev et al., 2006). Additionally, differences in CNV activation between younger and older adults suggest changes in response preparation processes (Falkenstein et al., 2002; Golob et al., 2005; Sterr and Dean, 2008).

As positive effects of anodal tDCS are more likely to emerge when motor processes are less efficient at baseline, we examined the effects of anodal tDCS on both dominant and non-dominant motor cortices. In the present experiment, we expected that old adults would show improved motor performance on the cued go/nogo task and associated ERP components after anodal tDCS over the M1, and that the tDCS effect would be greater when applied over the non-dominant hemisphere. For both dominant and non-dominant hemisphere stimulation, the effect should be greater for the contralateral than the ipsilateral hand.

## Method

### Participants

Thirty-nine right-handed healthy older adults^2^ completed testing under anodal tDCS and sham stimulation in separate sessions. Due to excessive EEG artifact, two participants were removed from further analyses, resulting in a final sample of 37 participants (19 males, mean age 59.9 ± 10.9 years). Participants were screened and assessed for handedness, as reported in Experiment 1. Participants also completed the Montreal Cognitive Assessment (MoCA, McLennan et al., 2011) to screen against dementia (27.33 ± 0.31). Participants were randomly assigned to stimulation condition: 21 participants (12 males, mean age 58.8 ± 9.9 years) received anodal tDCS over their dominant motor area, whereas the remaining 16 participants (7 males, mean age 61.2 ± 12.2 years) received active tDCS over their non-dominant motor area. Participants in both groups were randomly assigned to stimulation order as described in Experiment 1. This study was approved by the University of Newcastle’s Human Research Ethics (H-2010-1339).

### Design and Procedure

The parameters of the tDCS stimulation, the cued go/nogo paradigm and EEG recording were identical to those reported in Experiment 1, except as indicated below.

These older participants completed two tests of motor functioning often used clinically in stroke assessment, the JTT (Jebsen et al., 1969) and pinch grip tests (Hinson and Gench, 1989) both prior to and following tDCS intervention. In the cued go/nogo paradigm, the target-cue interval between trials was extended to accommodate slower RT in older adults (mean 3000 ms, random sequence, 2500–3500 ms). The statistical analyses of both the behavioral and the electrophysiological data included the between subjects factor: Stimulation Hemisphere (dominant vs. non-dominant). Target-locked P300 amplitude was estimated across a 250–650 ms interval.

## Results

### Behavioral Results

Results of the JTT and the pinch-grip tasks are presented in Marquez et al. (2015). For the cued go/nogo task, error rate for go trials and false alarm rate for nogo trials were very low and not statistically analyzed (1.61% and 0.1%, respectively). As shown in Figure 2B, RT was 338 faster for directional than for non-directional cues (*F*_(1,35)_ = 144.04, *p* < 0.001; 490 vs. 568.2 ms). There was no main effect of response hand (*p* > 0.05) or hemisphere (*F* < 1).

There was also no main effect of stimulation (tDCS: 534.6 ± 11, Sham: 523.6 ± 10; *F*_(1,35)_ = 2.12, *p* > 0.1). However, there was a three-way interaction between stimulation, cue and hemisphere (*F*_(1,35)_ = 5.2, *p* = 0.03). However, simple effects analyses within dominant and non-dominant hemisphere group separately resulted in no main effect of stimulation or stimulation by cue interaction (both *p* > 0.1). As shown in Figure 2B, stimulation over the dominant hemisphere showed a tendency for tDCS to increase (rather than decrease) RT. Stimulation over the non-dominant hemisphere showed a similar tendency for non-directional cues, but a small trend for faster RT under stimulation than sham for directional cues.

### Electrophysiological Results

#### CNV

Cue-locked ERP waveforms for healthy older adults are shown in Figure 3B. Both groups developed a centrally-maximal CNV, that was larger for directional than non-directional cues (−5.9 vs. −4.3 μV; *F*_(1,35)_ = 12.8, *p* < 0.001). There was no effect of response hand or interaction between cue and response hand on CNV amplitude (both *F* <1).

Figure 3B shows that tDCS over the dominant hemisphere appears to have reduced the effect of cue type on CNV amplitude. However, statistical analyses showed that stimulation had no main effect or interaction with response hand or hemisphere (all *p* >0.05). Moreover, the direction of the effect is opposite to our prediction that stimulation would increase response preparation and hence result in greater CNV difference between directional and non-directional cues.

#### P300

The target-locked P300 was larger for non-directional cues compared to directional cues (14.6 vs. 17.2 μV; *F*_(1, 35)_ = 35.6, *p* < 0.001; Figure 3B). There was a main effect of response hand, which showed significantly larger amplitudes for left compared to right hand responses (*F*_(1, 35)_ = 4.3, *p* = 0.047). There was no main effect of stimulation on P300 amplitude or any interaction with other factors (all *p* > 0.7). P300 latency was not significantly affected by either cue or response hand (both *p* > 0.1). While there was no main effect of stimulation (*F*_(1, 35)_ < 1, *p* < 0.8), the 4-way interaction between hemisphere, stimulation, response hand and cue was significant (*F*_(1, 35)_ = 4.6, *p* = 0.04). However this interaction also did not survive correction in simple analyses within each group.

#### LRP

The target-locked and response-locked LRPs for the old adults (Figure 4B) showed a pattern similar to that in young adults. tLRP emerged earlier and rLRP had a shorter duration for directional than non-directional cues (340.1 vs. 381.3 ms; *F*_(1,35)_ = 35.36, *p* < 0.001; −118.6 vs. −163.5 ms; *F*_(1,35)_ = 34. 6, *p* < 0.001, respectively). There was no main effect of stimulation or interaction between stimulation and other factors for either tLRP or rLRP (both *p* > 0.2).

## Discussion

Both behavioral and ERP measures showed a similar pattern to that seen in young adults, with faster responding, larger CNV, smaller P300, earlier tLRP onset and later rLRP onset for directional than non-directional cues. Old adults were noticeably slower in RTs and P300 latencies than young adults (Tables 1, 4), consistent with a disruption of motor processes with increasing age. Nevertheless, again, we found no effect of anodal tDCS over M1 on behavioral performance, ERP or LRP waveforms that would be consistent with enhancement of motor processes.

## Bayesian Analysis

Across both experiments, we found no evidence that anodal tDCS over M1 has a beneficial effect on either behavioral performance or the morphology of response-related ERPs. However, frequentist statistics do not allow us to conclusively assert that anodal tDCS over M1 has no effect on either response speed or motor-related ERP components. To assess the strength of the evidence in favor of a beneficial effect of tDCS vs. the null effects model, we performed Bayesian model selection analysis separately for each experiment on the factorial analyses of variance for each of the major ERP components as well as for response speed for older adults. As in Conley et al. (2015), we used the default-prior method for linear models as defined by Rouder et al. (2012) to create Bayes factors for each possible model. Bayesian analysis was performed using the BayesFactor package in R (Morey and Rouder, 2013), assuming the default setting for the fixed-effect prior (*r* = 0.5).

For Experiment 1, Bayesian analysis for RT was reported in Conley et al. (2015) and showed that the null effects model was 11 times more likely to fit the data than the strongest model including stimulation. The strongest model for each ERP component (CNV and P300 amplitudes, target and rLRP latencies) included only cue as a factor. For each ERP component, the strongest model that included stimulation as a factor had Bayes factors that were at least four times smaller than the null effects model. Consistent with the RT results for the young adults reported in Conley et al. (2015), there is evidence for no beneficial effect of anodal tDCS over M1 on motor-related ERPs in healthy younger adults.

In Experiment 2, Bayesian analysis of RTs showed that the most likely model to predict the data had an effect of cue factor only. The strongest model that included stimulation was around two and a half times less likely to predict than the null effects model. As seen in Table 2, any effect of anodal tDCS stimulation on RT tended to be in the opposite direction than predicted (ie, a slowing effect). The strongest model to predict both CNV and P300 amplitude included both hemisphere and cue factors. The strongest models that included stimulation condition had Bayes factors that were seven and six times smaller than the null effects model for CNV and P300, respectively. The strongest model to predict each LRP component consisted of cue condition only, and the strongest models that included stimulation were five and six times weaker than the null effects models for the rLRP and tLRP, respectively. Thus, consistent with the findings in young adults, healthy old adults also showed no beneficial effect of anodal tDCS over M1 on ERPs.

## General Discussion

This study investigated the effects of anodal tDCS over M1 on ERPs related to response processes in healthy young and old adults. Despite clear evidence that the task produced the expected behavioral and ERP effects, there was no evidence for a beneficial effect of anodal tDCS over the dominant M1 (young and old groups) or the non-dominant M1 (old group) on either RT or motor-related ERP waveforms. In fact, Bayesian analysis showed support for no effect of anodal tDCS over the M1; the null effect of stimulation model was at least twice as likely to explain the data as the strongest model including stimulation as a factor.

It is possible that the absence of a significant effect of anodal stimulation over the M1 may be due to a number of specific parameters in this study. For instance, the absence of an effect of stimulation on RT may be due to a ceiling effect, as both younger and older adults showed very high performance (i.e., above 90% accuracy). However, this interpretation is unlikely to explain the absence of any effect on ERP components that represent response preparation, selection and activation processes, as these represent the effectiveness of the underlying motor processes, rather than the decision process itself. This is especially true for older adults who showed typical ageing effects on ERP and given the well documented decline in cognitive and neuromuscular functioning in healthy ageing (Raz et al., 2005; Wu and Hallet, 2005). We therefore conclude that anodal tDCS over M1 does not affect motor ERPs in either healthy younger or older adults.

Another factor that may have contributed to the null results is the delay between application of stimulation and onset of testing, which arose because of the need to set up the EEG recording. It could be argued that the 30–40 min delay may have abolished any effect of anodal tDCS over M1 on behavior or ERPs. However, this is unlikely. Firstly, Conley et al. (2015) found no effect on behavioral performance in young adults, even when the task commenced immediately after stimulation or was completed concurrently with stimulation. Previous studies suggest that the long stimulation session used here (20 min) should elicit sustained post-stimulation effects lasting a minimum of 1 h (Nitsche and Paulus, 2001). Indeed, studies that have observed enhanced ERPs following anodal tDCS over DLPFC (Kongthong et al., 2013; Lafontaine et al., 2013; Lapenta et al., 2014) have found sustained effects following even briefer stimulation sessions (e.g., 13–20 min). One of these studies applied tDCS concurrently with the EEG recording (Lafontaine et al., 2013). However, the other studies set up EEG recordings after tDCS and would have had similar delays between the cessation of stimulation and task performance (19 and 128 channel EEG systems for Kongthong et al., 2013; Lapenta et al., 2014 respectively). Additionally in Lapenta et al. (2014), participants also completed another assessment between tDCS and EEG setup. We conclude that it is unlikely that the delay between tDCS intervention cessation and task performance can account for the lack of stimulation effects.

A final potential contributor to the null effect may be the specific stimulation parameters. We chose stimulation parameters that are commonly used in studies applying anodal tDCS over M1 and that have been shown to enhance both motor excitability (Nitsche and Paulus, 2001) and gross motor performance (Boggio et al., 2006). Recent computational models of anodal tDCS over M1 using the same stimulation parameters show an elicited electrical field that spread across most of the frontocentral areas of the cortex (Miranda et al., 2013). This indicates that the current should spread over areas that are directly involved in the response processes required by the cued go/nogo task (Praamstra et al., 1996; Gomez et al., 2003). Additionally, previous research has produced effects on ERPs following anodal tDCS using 1 mA currents (Kongthong et al., 2013). Therefore, stimulation parameters are unlikely to account for null effects.

Finally, the absence of improvement in performance following anodal tDCS over M1 is consistent with a number of recent studies (Bortoletto et al., 2015; Montenegro et al., 2015). Over the last 5 years, an increasing number of studies have failed to show facilitation of performance following anodal tDCS over the M1, consistent with the increased interest in reporting null as well as positive results. Null effects have been observed in motor function (Wiethoff et al., 2014; Montenegro et al., 2015) and visuomotor tasks (Ambrus et al., 2016), in both healthy young (Pellicciari et al., 2013) and older adults (Lindenberg et al., 2013). Indeed, a recent meta-analysis found that, with the exception of TMS studies of motor output, there is little consistent evidence of facilitation of performance following anodal tDCS over M1 (Horvath et al., 2015). The present study provides additional evidence for null effects following anodal tDCS over M1, by showing that electrophysiological measures associated with motor preparation (CNV), response selection (tLRP) and response execution (rLRP) are not affected by anodal tDCS over the M1 in either young or old adults.

## Author Contributions

Data was collected by ACC and JLM. ACC, WRF and FK were involved with the processing and analysis of data. All authors contributed to the interpretation of the data as well as the writing and finalization of the manuscript.

## Funding

This research was supported by a University of Newcastle postgraduate student scholarship for ACC (Reference No. 3092298) and a National Stroke Foundation Australia grant to JLM.

## Conflict of Interest Statement

The authors declare that the research was conducted in the absence of any commercial or financial relationships that could be construed as a potential conflict of interest.

## Footnotes

^1^The behavioral results were presented in Experiment 1 of Conley et al. (2015). Here we report the behavioral data very briefly, to allow comparison with data from older adults in Experiment 2.

^2^Data from most of these participants contributed to Marquez et al.’s (2015) which focused on clinical measures of motor performance (e.g., JTT and grip tasks).
