# Corrigendum: No Effect of Anodal Transcranial Direct Current Stimulation Over the Motor Cortex on Response-Related ERPs during a Conflict Task

**DOI:** 10.3389/fnhum.2016.00584

**Published:** 2016-11-17

**Authors:** Alexander C. Conley, W. R. Fulham, Jodie L. Marquez, Mark W. Parsons, Frini Karayanidis

**Affiliations:** ^1^Functional Neuroimaging Laboratory, School of Psychology, Faculty of Science and IT, University of NewcastleNewcastle, NSW, Australia; ^2^Priority Research Centre for Stroke and Brain Injury, University of NewcastleNewcastle, NSW, Australia; ^3^Hunter Medical Research InstituteNewcastle, NSW, Australia; ^4^School of Health Sciences, Faculty of Health, University of NewcastleNewcastle, NSW, Australia; ^5^School of Medicine and Public Health, Faculty of Health, University of NewcastleNewcastle, NSW, Australia

**Keywords:** transcranial direct current stimulation, event-related potential, contingent negative variation, lateralized readiness potential, P300, aging

It has come to the authors' attention that there is an error in Figure 4. Specifically, the line colors used in the LRP waveforms are not consistent with what is indicated in the legend. The authors sincerely apologize for the error. This error has been corrected in the revised figure.

**Figure 4:**
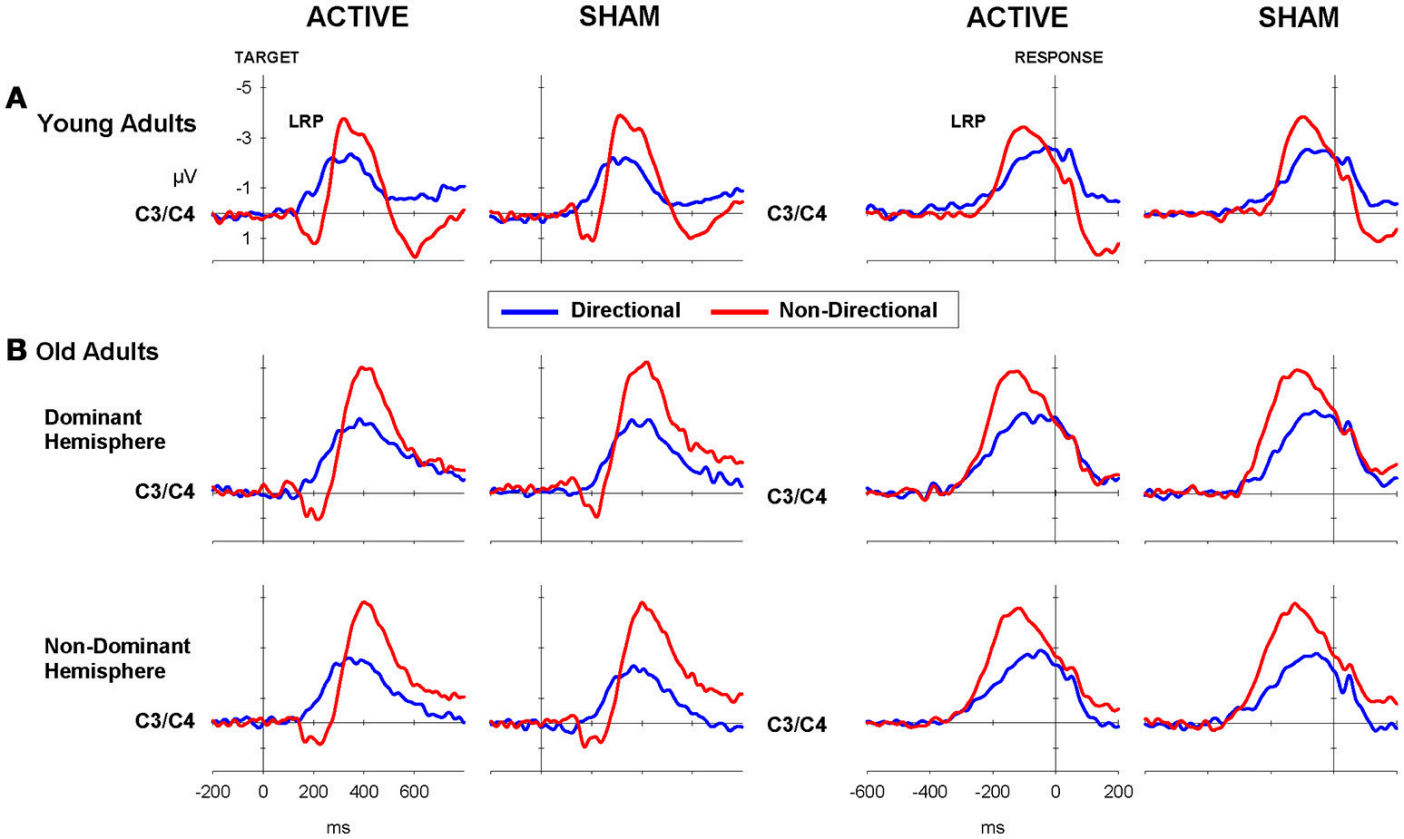
**LRP waveforms for directional (blue) and non-directional (red) conditions in target-locked (left) and response-locked (right) LRP waveforms following active (i.e., anodal tDCS) and sham for (A)** young and **(B)** old adults.

It is important to note that this error does not impact the conclusions of the article in any way, but may confuse readers as the direction of effects depicted in the original figure do not match the text.

## Conflict of interest statement

The authors declare that the research was conducted in the absence of any commercial or financial relationships that could be construed as a potential conflict of interest.

